# Challenges and Recommendations for Integrating Circadian Medicine in Critical Care: A Roadmap

**DOI:** 10.1016/j.chest.2025.12.010

**Published:** 2025-12-20

**Authors:** Floor W. Hiemstra, Liliana Bustos González, Lilian J. Engelhardt, Laura Hancke, Luisa K. Pilz, Amanda I. Adler, Hassan S. Dashti, Xavier Drouot, Gareth B. Kitchen, Melissa P. Knauert, Achim Kramer, Jonathan O. Lipton, Alawi Luetz, Matthew B. Maas, Nathan M. Pajor, Sairam Parthasarathy, Claudia Spies, David J. van Westerloo, Matthias Felten, Elizabeth B. Klerman, David W. Ray, Marc D. Ruben, Till Roenneberg, Laura Kervezee

**Affiliations:** a Department of Intensive Care, Leiden University Medical Center, Leiden, The Netherlands; b Group of Circadian Medicine, Department of Cell and Chemical Biology, Leiden University Medical Center, Leiden, The Netherlands; c School of Nursing, Faculty of Medicine, Universidad de Valparaíso, Viña del Mar, Chile; d Department of Anesthesiology and Intensive Care Medicine, Charité – Universitätsmedizin Berlin,corporate Member of Freie Universität Berlin and Humboldt-Universität zu Berlin, Berlin, Germany; e BIH Biomedical Innovation Academy, BIHCharité Junior Digital Clinician Scientist Program, Berlin Institute of Health at Charité – Universitätsmedizin Berlin, Berlin, Germany; f ECRC Experimental and Clinical Research Center, Charité – Universitätsmedizin Berlin, corporate member of Freie Universität Berlin and Humboldt-Universität zu Berlin, Berlin, Germany; g Oxford Centre for Diabetes Endocrinology and Metabolism, University of Oxford, Oxford, United Kingdom; h Department of Anesthesia, Critical Care and Pain Medicine, Massachusetts General Hospital, Boston, MA; i University Hospital of Poitiers, Poitiers, France; j Division of Immunology, Immunity to Infection and Respiratory Medicine, University of Manchester, Manchester, United Kingdom; k Section of Pulmonary, Critical Care and Sleep Medicine, Department of Internal Medicine, Yale School of Medicine, New Haven, CT; l Laboratory of Chronobiology, Charité–Universitätsmedizin Berlin, corporate member of Freie Universität Berlin, Humboldt-Universität zu Berlin, and Berlin Institute of Health, Berlin, Germany; m Division of Sleep Medicine, Harvard Medical School, Boston, MA; n Department of Neurology and Kirby Neurobiology Center, Boston Children’s Hospital, Boston, MA; o Department of Healthcare Management, Technische Universität Berlin, Berlin, Germany; p Department of Neurology, Northwestern University, Chicago, IL; q Department of Anesthesiology, Northwestern University, Chicago, IL; r Division of Pulmonary Medicine, Cincinnati Children’s Medical Center, Cincinnati, OH; s Division of Biomedical Informatics, Cincinnati Children’s Medical Center, Cincinnati, OH; t James M. Anderson Center, Cincinnati Children’s Medical Center, Cincinnati, OH; u Department of Pediatrics, University of Cincinnati College of Medicine, Cincinnati, OH; v University of Arizona Center for Sleep Circadian and Neuroscience Research, University of Arizona, Tucson, AZ; w Department of Infectious Diseases, Respiratory Medicine and Critical Care, Charité–Universitätsmedizin Berlin, corporate member of Freie Universität Berlin and Humboldt-Universität zu Berlin, Berlin, Germany; x Department of Neurology, Massachusetts General Hospital, Boston, MA; y NIHR Oxford Health Biomedical Research Centre, Oxford, United Kingdom; z Oxford Biomedical Research Centre, John Radcliffe Hospital, Oxford, United Kingdom; aa Oxford Kavli Centre for Nanoscience Discovery, University of Oxford, Oxford, United Kingdom; ab Chronsulting, Peterskirchen, Germany; ac Institute for Medical Psychology, LMU Munich, Munich, Germany; ad Institute and Polyclinic for Occupational, Social, and Environmental Medicine, LMU Munich, Germany

**Keywords:** circadian rhythms, critical care, critical illness, ICU, meeting report

## Abstract

**Background:**

Circadian rhythms are often severely disrupted in critically ill patients in the ICU. The ICU environment, characterized by irregular light-dark signals, continuous nutrition, and round-the-clock interventions, contributes to this disruption by providing weak and conflicting timing cues to the circadian system. Extensive scientific research has demonstrated that circadian rhythms play a vital role in regulating physiology and maintaining overall health. Therefore, integrating circadian principles into critical care may represent a promising strategy to improve patient outcomes in the ICU.

**Research Question:**

What are the key challenges of integrating circadian medicine into critical care, what steps can address these challenges, and which recommendations can guide future study designs and clinical implementation?

**Study Design and Methods:**

We convened a 5-day workshop in September 2024 that brought together 24 international experts with backgrounds in circadian biology, critical care, and implementation science. Each day was organized around a predefined theme, with morning presentations and plenary discussions, and afternoons dedicated to drafting a list of Propositions and Recommendations in breakout groups. Propositions and Recommendations were finalized via a post-workshop survey requiring ≥ 75% agreement.

**Results:**

This roadmap summarizes the discussions and outcomes of the workshop, structured around a set of Propositions and Recommendations, and provides a framework for building a robust evidence base for integrating circadian principles into ICU practice. Key recommendations include the development of circadian outcome measures tailored for use in the ICU and using standardized frameworks for evaluating the effect of circadian interventions in clinical trials.

**Interpretation:**

Altogether, this roadmap provides an interdisciplinary framework resulting from a collaborative effort of ICU clinicians, circadian biologists, and implementation specialists, for building a robust evidence base for integrating circadian principles into ICU research and practice.

**Take-Home Points**

**Research Question:** What are the key challenges of integrating circadian medicine into critical care, what steps can address these challenges, and which recommendations can guide future study designs and clinical implementation?

**Results:** This report summarizes the discussions and outcomes of a workshop focused on circadian biology in critical care, in which ICU clinicians, circadian biologists, and implementation specialists collaboratively developed a set of Propositions and Recommendations for integrating circadian principles into ICU practice, including the development of circadian outcome measures tailored for use in the ICU and using standardized frameworks for evaluating the effect of circadian interventions in clinical trials.

**Interpretation**: This roadmap provides a framework for building a robust evidence base for integrating circadian principles into ICU practice.

ICUs provide specialized care to critically ill patients around the clock. This continuous care often removes a daily structure from this environment: light levels are low during the day, darkness is replaced by dim light at night, nutrition is often provided across all 24 hours, social interactions occur any time, and physical activity and posture changes are minimal (Fig 1). While essential for ICU operations, these conditions, combined with disease-related pathophysiology, may disrupt the circadian coordination of physiology, potentially affecting clinical outcomes.^1^^,^^2^

**Figure 1:**
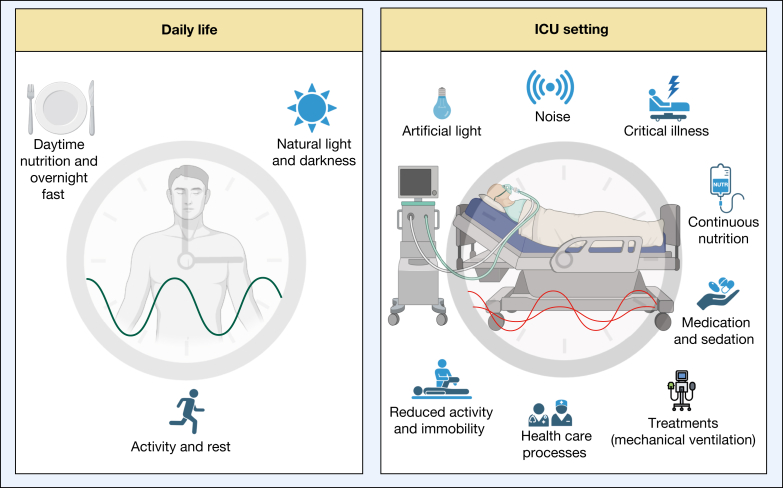
Timing cues for the circadian system. Comparison of the timing cues in out-of-hospital daily life (left panel) vs the weak and conflicting timing cues in the ICU (right panel).

In mammals, circadian coordination of physiology is influenced by the circadian system, which consists of a central clock in the suprachiasmatic nucleus of the hypothalamus and peripheral clocks in nearly all other tissues in the body. The suprachiasmatic nucleus is primarily synchronized by the light-dark cycle and conveys its rhythmicity to peripheral clocks throughout the body via neural and humoral signals.^3^ Almost all physiological processes are influenced by the circadian system, including the sleep-wake cycle, metabolism, hormone secretion, immune function, cardiovascular processes, cognitive function, and mood.^3^^,^^4^

Circadian rhythms, by definition, are self-sustained rhythms with a periodicity of approximately 24 hours that synchronize (entrain) to environmental timing cues (Zeitgebers). However, in real-world clinical settings, environmental or behavioral factors (eg, mobilization, drug administration, nutritional support) can contribute to 24-hour variations in physiology.^5^^,^^6^ Therefore, not all 24-hour rhythms are necessarily “circadian,” and we refer to these as “daily rhythms” in this article. In other contexts, we use “circadian” to refer broadly to principles related to the circadian system (eg, circadian interventions, circadian medicine).

The weak and conflicting input provided to the circadian system by the ICU environment may result in misalignments between the circadian system and the environment. Circadian misalignment for a few days can lead to increased BP, elevated levels of inflammatory markers, impaired immune function and glucose metabolism, decreased cognitive function, and sleep disturbances, even in healthy individuals.^7^, ^8^, ^9^, ^10^, ^11^ In critical illness, daily rhythms are often disrupted, which is associated with changes in clinical outcomes.^12^, ^13^, ^14^, ^15^

Findings from research on circadian biology are increasingly being incorporated into health care, including prevention, diagnosis, and therapy, giving rise to the field of circadian medicine.^16^, ^17^, ^18^, ^19^, ^20^ One hypothesis of circadian medicine is that strengthening and/or restoring circadian rhythms improves patient outcomes and supports recovery.^2^ Evidence from randomized controlled trials (RCTs) testing the potential benefit of circadian interventions in the ICU, however, remains inconclusive.^21^, ^22^, ^23^ Therefore, more robust study designs and better-defined outcome measures are warranted. Incorporating circadian research into critical care can also be used to find the optimal time(s) of day at which the efficacy of interventions is increased and/or the side effects are reduced.^24^ Promising targets include medications,^25^^,^^26^ ventilation,^27^ transplantation,^28^ and cardiac surgery,^29^ although more evidence is needed to fully understand the optimal timing and effectiveness of these interventions in clinical practice.

To chart the path from the promise of circadian medicine in critical care to measurable benefits, we organized a 5-day workshop in September 2024 at the Lorentz Center in Leiden (The Netherlands) attended by international experts with backgrounds in circadian biology, intensive care, and implementation science. The goals of the workshop were to: (1) develop a common understanding of the key research challenges in this field; (2) identify the steps needed to address these challenges; and (3) define a set of recommendations to guide future study designs and clinical implementation. This roadmap summarizes the discussions of the workshop along with a set of Propositions and Recommendations that were formulated during the workshop.

## Study Design and Methods

### Workshop Preparation

The workshop was prepared over the course of 9 months by an organizing committee (M. F., E. B. K., D. W. R., T. R., and M. D. R. chaired by L. K.), who met regularly online to shape the content (*Themes*) and organization (*Workshop structure*) of the meeting. The resulting in-person workshop (“About Time: Circadian Medicine in Critical Care,” September 16-20, 2024, at the Lorentz Center in Leiden, The Netherlands) convened 24 international experts. The organizing committee aimed for a balanced representation of expertise, gender, career stage, and international location. All participants contributed to drafting the manuscript and comprise the group of co-authors.

### Themes

Themes were defined prior to the meeting based on extensive discussion among the organizing committee. The five themes were: (1) evaluating the evidence base for circadian medicine in the ICU; (2) measures of circadian system function; (3) clinical outcome variables in circadian intervention studies; (4) design of circadian intervention studies; and (5) dissemination and implementation strategies.

The goal of the first theme was to identify key research challenges, with speakers selecting and presenting relevant content and discussions among participants who contributed their expertise and previous knowledge to identify gaps in the field. The other 4 themes focused on the steps needed to address these challenges and formulating recommendations for future research and clinical practice.

### Workshop Structure

Workshop mornings were dedicated to 1 of the 5 themes listed above, each chaired by a different member of the organizing committee. Each morning, the chair introduced the theme, after which 3 to 4 participants gave a short presentation on a subtheme, followed by a plenary discussion. Speakers were assigned to a specific subtheme based on their expertise in that area and were asked to cover 3 main points: (1) what is known; (2) evidence gaps; and (3) concrete steps and recommendations to guide future studies and clinical implementation. All presentations were submitted to the organizing committee in advance and reviewed for content, relevance, and overlap.

Workshop afternoons were dedicated to formulating recommendations and drafting of the manuscript in break-out sessions. Each break-out group consisted of the theme chair and a note-taker, along with two to three participants rotating between groups to ensure that each participant contributed to multiple themes. Workshop days concluded with summaries and progress discussions. In addition to this formalized structure, a representative from IC-Connect, the Dutch ICU patient organization, shared their experience as a former ICU patient.

### Definition of Propositions and Recommendations

Following the workshop, the workshop coordinator drafted a preliminary list of Propositions (Theme 1) and Recommendations (Themes 2-5) that reflected the content of the discussions during the plenary and break-out sessions during the workshop. The list of Propositions and Recommendations was circulated among all other participants, who were asked via an online survey to (1) indicate whether they agree with each item or not; (2) provide suggestions to improve the items by rephrasing; and (3) rate the importance of each item. Finally, the participants were asked whether they thought the list of items reflected the key points and scope of the discussions during the workshop. A 75% agreement rate was predefined as the threshold to confirm that a Proposition or Recommendation would be retained. After the survey, note-takers revised the Propositions and Recommendations based on participant open-ended feedback in a single iteration. The formulated Propositions and Recommendations, along with responses from the survey, were used to prepare the following sections of the manuscript.

## Results

### Results From Survey on Propositions and Recommendations

**Figure 2:**
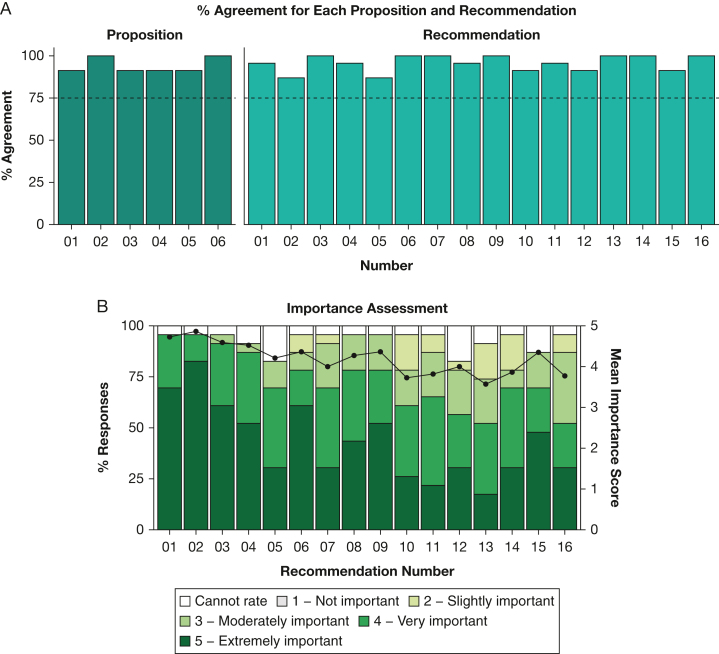
A, B, Survey results. A, Percentage of participants that agreed with each item in the survey. The black dotted line represents the pre-defined threshold of 75% used to determine whether each Proposition or Recommendation would be retained or discarded. Participants provided open-ended feedback to improve the wording of each item in the survey. B, Importance assessment of each Recommendation using a 1 to 5 scale. The mean importance score (right y-axis) is calculated as the mean score across all participants. Numbers of Propositions and Recommendations in this figure refer to the numbers of the Propositions and Recommendations in Figures 3 and 4, respectively.

**Figure 3:**
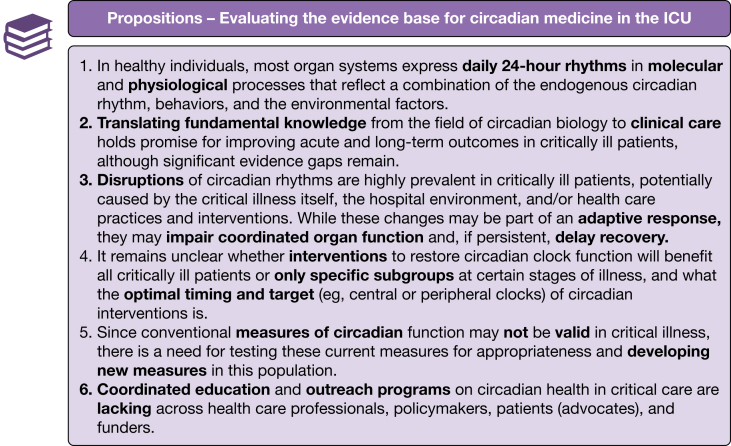
Roadmap for circadian medicine in the ICU summarizing the Propositions formulated during the workshop. These Propositions represent the final consensus after iterative refinement and approval by the expert panel.

**Figure 4:**
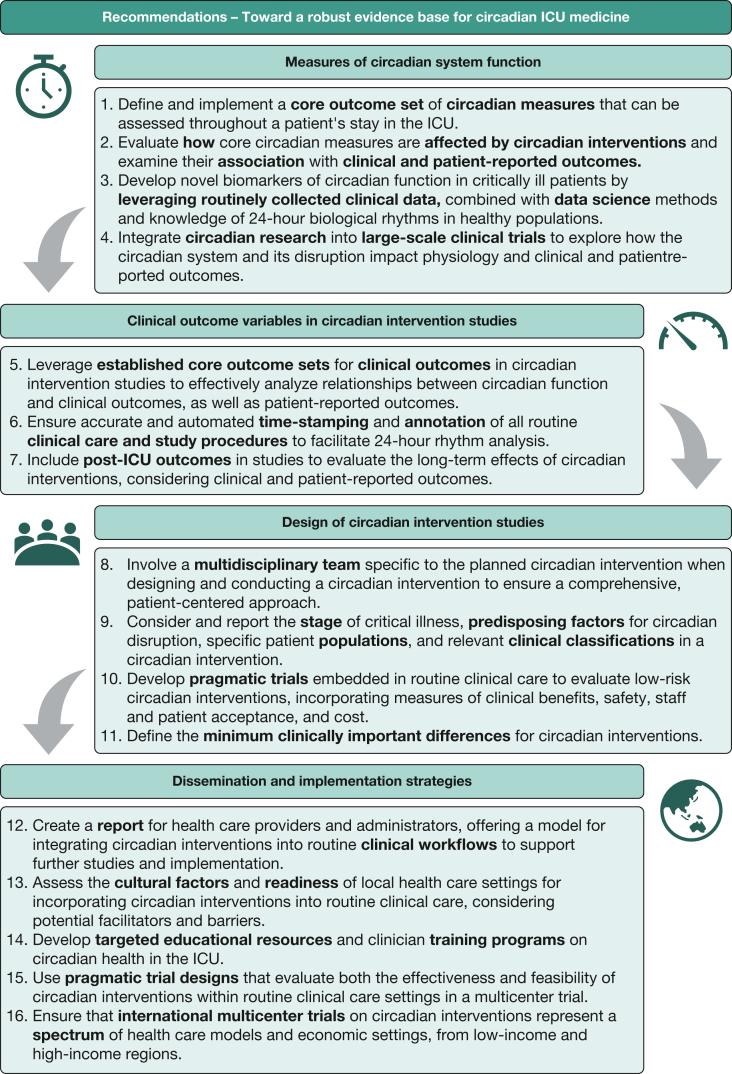
Roadmap for circadian medicine in the ICU summarizing the Recommendations formulated during the workshop. These Recommendations represent the final consensus after iterative refinement and approval by the expert panel.

The list of initial Propositions and Recommendations used in the online survey is provided in e-Tables 1 and 2. All participants except the workshop coordinator completed the survey (n = 23). For all Propositions and Recommendations, the percentage of participants that expressed agreement exceeded the predefined threshold of 75% (median, 96%; range, 87%-100%) (Fig 2A), so all items were included in the final list of Propositions (Fig 3) and Recommendations (Fig 4). The average importance rating of the Recommendations ranged from 3.6 to 4.9 on a 5-point scale (Fig 2B). All participants agreed that the list of Propositions and Recommendations accurately represents the main points and scope of the workshop discussions (e-Table 3). In the following sections, we describe these Propositions and Recommendations in more detail and provide the necessary context.

### Theme 1: Evaluating the Evidence Base for Circadian Medicine in the ICU

Preclinical and clinical evidence links disrupted circadian rhythms to impaired physiology (eg, immunologic, metabolic, neurologic, cardiovascular disorders).^30^ Nevertheless, this knowledge has not yet permeated ICU practice: many research challenges remain, as highlighted in a statement by the American Thoracic Society.^31^ These challenges have to be addressed for circadian medicine to realize its potential for improving acute and long-term outcomes in critically ill patients and critical illness survivors (Propositions 1 and 2) (Fig 3).

Although it is known that daily rhythms are attenuated during the acute stage of critical illness,^32^, ^33^, ^34^, ^35^ it is unclear to what extent an attenuation of rhythmicity is part of an adaptation to life-threatening conditions (ie, whether reducing circadian influence may improve patient outcomes under certain circumstances) (Proposition 3). Theoretically, acutely suppressing circadian regulation could be a strategy to conserve energy or maintain a continuous, robust immune response. If attenuating or changing circadian regulation is beneficial in some conditions, the key question would be *when* it becomes maladaptive. Analogous to stress responses, an attenuation or change of rhythmicity may be advantageous only at an initial, acute stage, while systemic and prolonged responses exact a toll that increases risk for longer term negative outcomes. Addressing this question is fundamental, given that interventions targeting or exploiting the circadian system will be meaningful only when the clock is functionally relevant. Therefore, it is important to understand how critical illness affects circadian control of physiology and under what conditions exposing ICU patients to circadian interventions is indeed beneficial or potentially disadvantageous.

To advance our understanding of whether a functional circadian system is advantageous, we need to learn more about the causes that contribute to the disruption of daily physiological rhythms in the ICU. Changes in daily rhythmicity may be integral to the critical illness itself, an adaptation to the ICU environment (eg, lighting or noise), and/or may result from treatments (eg, continuous nutrition, sedatives).^13^^,^^36^ The effectiveness of circadian interventions may also depend on who receives them, when they are introduced during the course of critical illness, the characteristics of the condition at that time, and what part of the circadian system (central clock and/or peripheral clocks) is targeted (Proposition 4). The extreme heterogeneity of ICU patients may affect any intervention’s effectiveness at the population level. For example, the evidence for melatonin and melatonin receptor agonists in reducing the risk of delirium is heterogeneous and varies across study populations.^23^^,^^37^, ^38^, ^39^ An extra layer of complexity is added when considering circadian interventions in neonatal ICU or PICU populations, since the exact developmental trajectory of daily rhythms in physiological and behavioral processes is still unknown.^40^^,^^41^

To address these evidence gaps, we need to define which physiological markers can be used to monitor circadian system function, specifically in the ICU (Proposition 5). There is extensive knowledge on how to derive such markers in healthy individuals and in controlled settings. Further investigation is required to determine which markers can reliably monitor circadian system function in critically ill patients. For example, daily cortisol rhythms may be obscured by steroid administration or stress-axis activation, and melatonin levels may be elevated due to decreased metabolism in patients with liver failure^42^ or by adrenergic drug-induced melatonin synthesis.^43^ Methods proposing circadian phase estimation using RNA expression analysis in healthy individuals^44^^,^^45^ are expected to be limited (or biased) by disrupted daily rhythm patterns in gene expression in critically ill patients.^33^^,^^34^^,^^46^ Metrics derived from actigraphy, often used as proxies for circadian function,^47^^,^^48^ are less reliable in immobile or sedated patients, or those with ICU-acquired weakness. Metrics of daily rhythmicity in vital signs (eg, heart rate, BP, core body temperature) have been used to study rhythm disruption and association with clinical outcomes on a large scale,^12^^,^^49^, ^50^, ^51^, ^52^ but standardized analysis frameworks to compute these metrics are lacking. Notably, vital sign fluctuations are influenced not only by circadian variation but also by environmental and behavioral factors.^5^^,^^6^ For example, events such as atrial fibrillation, vasopressor administration, and extracorporeal membrane oxygenation can significantly alter heart rate, BP, and body temperature, reducing the reliability of daily rhythm markers as indicators of actual circadian rhythms.

To successfully fill the evidence gaps, we need a cohesive network of relevant professionals (eg, ICU clinicians as well as researchers with expertise in circadian biology and implementation science). Structured education and outreach programs that target health care professionals, policymakers, patients (advocates), and funders to raise awareness of the topic are also crucial. These efforts are essential to foster stronger collaboration and ensure multidisciplinary input, which is the base to build a robust evidence base (Proposition 6).

Having identified the key evidence gaps, the workshop participants then focused on defining recommendations to address these gaps (Themes 2-5).

### Theme 2: Measures of Circadian System Function

Our first Recommendation is to define and implement a “core outcome set” of circadian measures (circadian COS) that can be assessed throughout a patient's stay in the ICU (Recommendation 1) (Fig 4). A COS is a standardized set of outcomes to be measured and reported for clinical trials in a specific area of health care, improving consistency, reliability, and comparability of results.^53^ The circadian COS would specify the measured physiological processes and statistical method(s) used to estimate one or more key rhythmic properties: period, amplitude, and/or phase (timing). Relevant physiological processes may include vital signs (eg, core body temperature, heart rate, BP), hormones (eg, melatonin, cortisol), expression of clock genes, or transcriptome-wide profiling. Clear guidelines for sampling frequencies and analysis methods, such as cosinor and spectral analysis, or other appropriate (parametric or non-parametric) methods, should be included. The development of this circadian COS should be consensus-driven,^54^ in which the biological relevance, degree of evidence, as well as the reliability and feasibility in the ICU setting, are carefully considered.

A circadian COS would allow rigorous evaluation of how the circadian system, both central and peripheral clocks, responds to interventions in the ICU such as dynamic lighting schedules or time-restricted feeding and how they are associated with clinical and patient-reported characteristics and outcomes (Recommendation 2). Widespread implementation of the circadian COS would provide standardized distributions of its variables in different ICU populations, and subgroups, allowing consistent circadian phenotyping of critically ill patients. Importantly, this approach may also help to elucidate how critical illness or specific pathological conditions contribute to circadian disruption.

We also recommend exploiting the wealth of clinical data routinely collected in the ICU^5^^,^^55^ such as vital signs (eg, core body temperature, BP, and heart rate [including its variability]), laboratory values, and the timing of treatments and other health care processes.^52^^,^^56^^,^^57^ This will allow the assessment of daily rhythms on a large scale, contributing to the development of novel biomarkers of circadian system function. Advanced data science methods (eg, machine learning, time series analysis) and benchmarking against healthy populations would greatly enhance the understanding of circadian system function in ICU populations (Recommendation 3). An important step in these analyses will be the handling of confounders, including health care processes, (dynamic) health conditions or status, or ongoing interventions that influence or mask the circadian measure of interest. Collecting, storing, structuring, annotating, analyzing, and interpreting those data will require expertise from an interdisciplinary team.

Finally, we recommend incorporating circadian research (eg, concepts and measures) into large-scale clinical trials to explore how the circadian system and its disruption affect physiology and clinical outcomes (Recommendation 4). This can be achieved by including targeted daily rhythm assessment (eg, hormone or clock gene measurements) in a subset of patients in large trials, or by analyzing daily rhythms of routinely collected data, such as vital signs, as exploratory end points. This provides valuable mechanistic insight with minimal logistical or financial burden, while still maintaining or improving statistical power for primary outcomes.

### Theme 3: Clinical Outcome Measures in Circadian Intervention Studies

Since the circadian system influences nearly all aspects of physiology, circadian interventions may impact a wide range of clinical outcomes.^1^ Existing circadian intervention studies have focused on outcomes such as melatonin rhythms,^36^^,^^58^ sleep quality,^59^ nutritional outcomes,^60^ and delirium risk.^21^^,^^36^^,^^59^^,^^61^ The connection between circadian rhythms and meaningful and patient-centered outcomes such as duration of mechanical ventilation or ICU stay or cognition in survivors is still underdeveloped and requires more observational and intervention-based research. Outcome measures must be prespecified and mechanistically justified based on the intervention’s hypothesized circadian targets, the study population, and the trial context.

To build a robust evidence base that informs clinical practice about the efficacy of circadian interventions, we encourage using appropriate established COSs for clinical outcomes that are available for many ICU-related outcomes^62^ (Recommendation 5). This includes delirium incidence,^63^ duration of mechanical ventilation,^64^ nutritional and metabolic interventions,^65^ physical rehabilitation,^66^ and perioperative infection and sepsis.^67^ Furthermore, given its close connection to circadian function, sleep should be considered as a relevant outcome, assessed either objectively (eg, polysomnography) or subjectively (eg, sleep questionnaires, such as the Richards-Campbell Sleep Questionnaire in adult ICU patients,^68^^,^^69^ or observational sleep stage classification for preterm infants in the neonatal ICU^70^). Given the potentially positive effects of circadian interventions on developmental outcomes that have been observed in preterm infants in the neonatal ICU,^71^ we also recommend circadian intervention studies involving pediatric populations to consider inclusion of developmental outcomes.

Accurate time-stamping is essential because interpretation of results depends on precise temporal annotation of the data. The timing of events (eg, the administration of medication, initiation and weaning of mechanical ventilation, or nutrition therapy) and environmental influences (eg, changes in exposure to noise or light, such as phototherapy in the neonatal ICU^72^) provides critical context for understanding daily physiological rhythms and their confounders in critical illness. We therefore recommend standardized and automated time-stamping and annotation of all routine clinical care and clinical trial procedures (Recommendation 6).

Recovery extends beyond ICU discharge; many patients experience persistent disruptions in daily rhythms and sleep patterns after transition from the ICU to another ward or discharge from the hospital.^73^^,^^74^ We recommend systematic inclusion of post-ICU outcomes in circadian intervention studies (Recommendation 7).

### Theme 4: Design of Circadian Intervention Studies

One application of circadian medicine in the ICU involves both creating a daily structure and optimizing treatments based on time-of-day-dependent effects.^75^^,^^76^ From a circadian medicine perspective, the daily ICU structure may include modifications of light conditions, timed feeding and mobilization, or timed scheduling of interventions and medications. Dedicated trials are required to study their clinical and cost-effectiveness, feasibility, safety, and acceptance, while considering contamination bias by unintended spillover of the intervention to patients who serve as controls in the study. During trial design and execution, a multidisciplinary team should be involved such as clinicians (eg, physicians, nurses, dietitians, physiotherapists, respiratory therapists, psychologists), patients and/or family advocates, basic scientists, pharmacists, data scientists, statisticians, public and economic health specialists, technicians, and others (Recommendation 8). In addition, it is important to consider individual preferences of patients and family members when planning and conducting a trial to assess a circadian intervention (eg, light sensitivity, eating and sleeping habits/schedules) to ensure a patient-centered approach.

Since the interaction between the circadian system and critical illness may change over the ICU stay, with potential implications for the effectiveness of a given intervention, we recommend carefully considering and documenting the stage of critical illness in the design of studies (Recommendation 9). For example, if attenuated circadian rhythmicity is an adaptive mechanism during early stages of critical illness, interventions may be more effective at later stages, and may therefore be beneficial only when they are initiated later in the ICU stay or continued on other hospital wards. Of note, follow-up of a circadian intervention trial may also need to extend beyond the hospital stay to study effects on post-ICU outcomes, such as post-intensive care syndrome. Likewise, documentation of clinical classifications and predisposing factors for circadian disruption is important. For example, some circadian interventions may not be effective in deeply sedated patients.

Novel methodological approaches to clinical trial design provide an opportunity for circadian medicine in the ICU. It is increasingly recognized that traditional RCTs, with strict eligibility criteria and highly controlled conditions, have limitations in terms of generalizability, heterogeneity, and costs.^77^^,^^78^ The nonintrusive and low-risk nature of some circadian interventions (eg, lighting and nutrition) makes them well-suited to be studied in *pragmatic* clinical trials, in which an intervention is tested to include in routine care.^79^, ^80^, ^81^ In such trials, recruitment, screening, and collection of outcome measures are automated as much as possible to reduce the financial and logistical burden of the trial, while still using randomization to assign patients or clusters to the treatments or conditions of interest.^79^^,^^80^ Pragmatic trials can increase both generalizability and efficiency compared with traditional RCTs. We encourage developing pragmatic trials to evaluate the effect of circadian interventions in critical illness (Recommendation 10). These trials should include measures of clinical effectiveness, safety measures, acceptance by staff and patients, and cost.

When performing an RCT is not feasible, analyses of non-randomized or non-interventional data including target trial emulation may be an alternative to study circadian interventions. Target trial emulation involves using observational data to simulate the conditions of a target trial (ie, a hypothetical RCT).^82^^,^^83^ Target trial emulation can help to define the minimal clinically important difference for a circadian intervention compared to standard care (ie, the smallest change in an outcome that is considered clinically relevant) (Recommendation 11).^84^ An example of a circadian intervention that is well-suited for target trial emulation is a light intervention using built-in lighting systems. If these systems are available only in a limited number of rooms, randomization would be inappropriate. While still allowing for causal inference, target trial emulation would support rigorous evaluation of this intervention in real-world conditions.

### Theme 5: Dissemination and Implementation Strategies

Careful reporting of implementation strategies and outcomes of circadian interventions in the ICU is critical to translate their potential into practice, as implementation will present unique challenges. Specifically, practical barriers include staff issues, such as the perceived increase in nursing workload; significant financial costs associated with technologies such as dynamic lightning; and institutional culture, particularly the clinical inertia against altering established 24/7 routines. These challenges may limit acceptance among health care teams. Therefore, we suggest creating guidelines for health care providers and administrators that offer a model for integrating any circadian interventions that are shown to be effective in RCTs into routine clinical workflows (Recommendation 12). Interprofessional communication frameworks may be helpful in evaluating the implementation of circadian conditions during daily patient rounds.^85^ For this reason, it is important to assess cultural factors and the readiness of local health care institutions to adopt these measures in routine clinical practice (Recommendation 13). This includes identifying facilitators and potential barriers to adopting effective circadian interventions. The development of targeted educational resources and training programs for ICU staff on the importance of the circadian system for patient health and recovery can also play a key role in overcoming resistance (Recommendation 14).

Robust evidence on the impact of circadian conditions in clinical practice is needed to help clinical teams and financial decision makers evaluate the benefits. We therefore recommend pragmatic, multicenter trial designs of circadian interventions that have shown promise in small-scale studies to assess effectiveness and feasibility within routine clinical care in different clinical settings (Recommendation 15). Finally, it is important to include centers from different health care models and economic settings, from both low-income and high-income regions, to capture a wide range of exposures and ensure broad applicability of the results (Recommendation 16).

## Discussion

Patients in the ICU may experience severe disruption of their circadian system, which may negatively affect health outcomes and recovery. Creating an environment that supports circadian system function at appropriate times therefore holds great potential for improving meaningful patient outcomes. However, effective integration of circadian biology in the ICU remains limited until well-designed studies demonstrate whether interventions targeted at the circadian system reduce circadian disruption and whether this improves health outcomes. In our workshop, we identified key evidence gaps, and we propose a set of recommendations to address these challenges and guide future research and clinical practice. Key recommendations include the development of circadian outcome measures tailored for use in the ICU and using standardized frameworks and COSs for evaluating the effect of circadian interventions in clinical trials.

Several limitations of this work should be acknowledged. First, we did not follow a standardized consensus methodology, such as a Delphi process, which may have limited the systematic assessment of agreement and prioritization across participants. Nevertheless, a structured survey was conducted to collect input and arrive at a final set of Propositions and Recommendations, and all participants accepted an invitation to be co-authors of the manuscript. Second, the workshop presentations were prepared by the speakers themselves and, although reviewed by the organizing committee for relevance and content, no formal assessment of potential bias was conducted.

Despite these limitations, the workshop has important strengths. Participants represented a multidisciplinary group, including ICU clinicians, circadian biologists, implementation specialists, and patient representatives. This diversity of expertise enabled comprehensive discussions, ensured multiple perspectives were considered, and strengthened the relevance and applicability of the resulting Propositions and Recommendations. Finally, we did not conduct a de novo systematic review and meta-analysis but built upon expert consensus following review of the body of scientific literature in this field. We recognize that a systematic review and meta-analysis is more robust, but considering the vast methodological and conceptual diversity of this nascent research area, we resorted to an expert consensus whilst recognizing the inherent bias of such an approach.

## Interpretation

Through a structured and collaborative effort of ICU clinicians, circadian biologists, and implementation specialists, the workshop led to a set of Propositions and Recommendations that will help to translate knowledge on the circadian system to the field of critical care. Thereby, this roadmap provides a framework for building a robust evidence base for integrating circadian principles into ICU practice.

## Funding/Support

The workshop was supported by the 10.13039/501100014102Lorentz Center [https://www.lorentzcenter.nl/] and the BioClock Consortium [https://bioclockconsortium.org/] funded by the research program NWA-ORC of the Dutch Research Council [https://www.nwo.nl/en; 1292.19.077 to L. K.]. This work was further supported by a VENI grant from the Netherlands Organisation for Health Research and Development (ZonMw) [https://www.zonmw.nl/en; 2020-09150161910128 to L. K.], an institutional project grant from the 10.13039/501100005039Leiden University Medical Center [https://www.lumc.nl/; to L. K. and D. J. v .W.], 10.13039/501100020884Agencia Nacional de Investigación y Desarrollo (ANID) Grant for Academic Installation [https://anid.cl; 2023 85220042 to L. B. G.], the 10.13039/501100000272National Institute for Health and Care Research (NIHR) Oxford Biomedical Research Centre [https://oxfordbrc.nihr.ac.uk, to A. I. A.; NIHR203316, to D. W. R.], 10.13039/100000002National Institutes of Health [https://www.nih.gov/; R00HL153795 to H. S. D.; HL151368 and NS126547 to J. O. L.; 1OT2HL156812-01 to M. B. M.], Broad/Boston Investment Conference [https://bostoninvestmentconference.com/; to J. O. L.], Murray and Clara Walker Endowment [https://www.asufoundation.org/education-and-scholarship/donor-named-funds/murray-and-clara-walker-memorial-scholarship-CA114070.html; to S. P.], Medical Research Council [https://www.ukri.org/councils/mrc/; MR/W019000/1 and MR/V034049/1 to D. W. R.], the NIHR Manchester Biomedical Research Centre [https://www.manchesterbrc.nihr.ac.uk/; NIHR203308 to G. B. K.], Research and Innovation, Manchester University NHS Foundation Trust [https://mft.nhs.uk/; to G. B. K.], and the NIHR Health Technology Assessment [https://www.nihr.ac.uk/research-funding/funding-programmes/health-technology-assessmen; NIHR156500 to G. B. K.].

## Financial/Nonfinancial Disclosures

The authors have reported to *CHEST* the following: X. D. reports a pending patent (a sleep analyzer and associated systems and methods) and ownership of stock option in Somno Engineering. A. K.’s institution has issued patents (PCT/EP2018/066771 and PCT/EP2022/087463) related to biomarkers for detecting the clock. This intellectual property has been licensed by BodyClock Technologies GmbH, in which A. K. is a shareholder. A. K. receives editor’s honoraria from Springer Verlag and Georg Thieme Verlag. E. B. K. consulted for Circadian Therapeutics, the National Sleep Foundation, and Yale University Press; received honoraria from the American Academy of Sleep Medicine Foundation, Sleep Research Society, and Sleep Research Society Foundation; and received travel support from the European Biological Rhythms Society, Lighten up/EPFL Pavilions, Sante Fe Institute, Sleep Research Society, Society for Research in Biological Rhythms, and World Sleep Foundation. E. B. K.’s partner is founder and CSO of Chronsulting. T. R. is owner, founder, and CSO of Chronsulting UG. None declared (F. W. H., L. B. G., L. J. E., L. H., L. K. P., A. I. A., H. S. D., G. B. K., M. P. K., J. O. L., A. L., M. B. M., N. M. P., S. P., C. S., D. J. v. W., M. F., D. W. R., M. D. R., L. K.).

## Supplementary Data

e-Online Data
