# Supplementary material for: Challenges and Recommendations for Integrating Circadian Medicine in Critical Care: A Roadmap
Source: Chest. 2025 Dec 20;169(4):977–90. doi: 10.1016/j.chest.2025.12.010 (PMC13083041; doi:10.1016/j.chest.2025.12.010)
Supplement: e-Online Data [file mmc1.pdf]

Online Data Supplement

Challenges and Recommendations for Integrating Circadian Medicine in Critical Care: A Roadmap

Hiemstra FW; Bustos González L; Engelhardt LJ; Hancke L; Pilz LK; Adler AI; Dashti HS; Drouot X; Kitchen GB; Knauert MP; Kramer A; Lipton JO; Luetz A; Maas MB; Pajor NM; Parthasarathy S; Spies C; van Westerloo DJ; Felten M; Klerman EB; Ray DW; Ruben MD; Roenneberg T; Kervezee L

Table E1. List of preliminary and final propositions. …………………………………………… 2

Table E2. List of preliminary and final recommendations. ……………………………………... 3

Table E3. Responses to question “Does this list of propositions and recommendations reflect the key points and scope of the discussions during the Lorentz workshop?”………………… 5

Table E1. List of preliminary and final propositions. These preliminary propositions were revised based on participants' feedback and reordered to fit the structure of the manuscript. The final versions are also presented in Figure 3 in the main manuscript.

	**Propositions**	
	Preliminary	***Final***
**1**	All major organ systems express daily 24-hour rhythms in molecular and physiological function that are regulated by the circadian timing system.	In healthy individuals, most organ systems express daily 24-hour rhythms in molecular and physiological processes that reflect a combination of the endogenous circadian rhythm, behaviors, and the environmental factors.
**2**	The field of circadian biology shows promise for improving clinical, patient-reported, and long-term outcomes in critically ill patients, although significant evidence gaps remain.	Translating fundamental knowledge from the field of circadian biology to clinical care holds promise for improving acute and long-term outcomes in critically ill patients, although significant evidence gaps remain.
**3**	Disruptions to these endogenous 24-hour rhythms are highly prevalent in critically ill patients, potentially caused by illness, the environment, and/or other interventions, which may impair organ function and recovery.	Disruptions of circadian rhythms are highly prevalent in critically ill patients, potentially caused by the critical illness itself, the hospital environment, and/or healthcare practices and interventions. While these changes may be part of an adaptive response, they may impair coordinated organ function and, if persistent, delay recovery.
**4**	It remains unclear whether interventions to restore circadian clock function will benefit all ICU patients or only specific subgroups at certain stages of illness.	It remains unclear whether interventions to restore circadian clock function will benefit all critically ill patients or only specific subgroups at certain stages of illness, and what the optimal timing and target (e.g., central or peripheral clocks) of circadian interventions is.
**5**	Since classical measures of sleep and circadian function are not always be adequate in critical illness, there is a need for new outcome measures to assess sleep and circadian clock function in this population.	Since conventional measures of circadian function may not be valid in critical illness, there is a need for testing these current measures for appropriateness and developing new measures in this population.
**6**	Coordinated education and outreach programs on sleep and circadian health in critical care are lacking across healthcare professionals, policymakers, patient advocates, and funders.	Coordinated education and outreach programs on circadian health in critical care are lacking across healthcare professionals, policymakers, patients (advocates), and funders.

Table E2. List of preliminary and final recommendations. These preliminary recommendations were revised based on participants' feedback and reordered to fit the structure of the manuscript. The final versions are also presented in Figure 4 in the main manuscript.

	**Recommendations**	
	Preliminary	***Final***
**1**	Develop and implement a set of core circadian outcome measures that can be assessed throughout a patient's stay in critical care.	Define and implement a core outcome set of circadian measures that can be assessed throughout a patient's stay in the ICU.
**2**	Evaluate how these core circadian outcome measures are impacted by circadian-based interventions (e.g., altered lighting and/or feeding schedules) and how they correlate with clinical or patient-reported outcomes.	Evaluate how core circadian measures are affected by circadian interventions and examine their association with clinical and patient-reported outcomes.
**3**	Leverage existing electronic health records (EHR) data, combined with data science methods and knowledge of 24-hour rhythms in healthy populations, to develop new biomarkers of circadian function in critically ill patients.	Develop novel biomarkers of circadian function in critically ill patients by leveraging routinely collected clinical data, combined with data science methods and knowledge of 24-hour biological rhythms in healthy populations.
**4**	Embed mechanistic (circadian) research inside large-scale clinical trials to explore how the circadian system and its disruption impact physiology & clinical outcomes.	Integrate circadian research into large-scale clinical trials to explore how the circadian system and its disruption impact physiology & clinical and patient-reported outcomes.
**5**	Make use of existing core outcome sets in circadian-based intervention studies to establish relationships with clinical & patient-centered outcomes.	Leverage established core outcome sets for clinical outcomes in circadian intervention studies to effectively analyze relationships between circadian function and clinical outcomes, as well as patient-reported outcomes.
**6**	Ensure accurate time-stamping of routine clinical care and study procedures.	Ensure accurate and automated time-stamping and annotation of all routine clinical care and study procedures to facilitate 24-hour rhythm analysis.
**7**	Consider inclusion of post-ICU outcomes to assess long-term effects of circadian-based intervention studies.	Include post-ICU outcomes in studies to evaluate the long-term effects of circadian interventions, considering clinical and patient-reported outcomes.
**8**	Involve a multi-disciplinary team when designing a trial that includes clinicians, circadian biologists, nurses, and patient representatives.	Involve a multidisciplinary team specific to the planned circadian intervention when designing and conducting a circadian intervention to ensure a comprehensive, patient-centered approach.
**9**	Consider the stage of critical illness (e.g., acute, sub-acute, chronic) and specific clinical subgroups when formulating eligibility criteria.	Consider and report the stage of critical illness, predisposing factors for circadian disruption, specific patient populations, and relevant clinical classifications in a circadian intervention.

**10**	Clearly define the comparative effectiveness of interventions (incl. benefit, harm, and costs).	Develop pragmatic trials embedded in routine clinical care to evaluate low-risk circadian interventions, incorporating measures of clinical benefits, safety, staff and patient acceptance, and cost.
**11**	Establish the minimum clinically important difference to measure the effectiveness of interventions.	Define the minimum clinically important differences for circadian interventions.
**12**	Report on how to integrate circadian-based interventions in routine clinical care to facilitate implementation.	Create a report for healthcare providers and administrators, offering a model for integrating circadian interventions into routine clinical workflows to support further studies and implementation.
**13**	Assess local culture and readiness for adopting changes related to sleep and circadian rhythms in clinical routines.	Assess the cultural factors and readiness of local healthcare settings for incorporating circadian interventions into routine clinical care, considering potential facilitators and barriers.
**14**	Develop educational resources and training programs about sleep and circadian health in the ICU.	Develop targeted educational resources and clinician training programs on circadian health in the ICU.
**15**	Make use of pragmatic trial designs that embed interventions within routine clinical care, so the effectiveness and feasibility of interventions can be studied on a large scale in multi-center trials.	Use pragmatic trial designs that evaluate both the effectiveness and feasibility of circadian interventions within routine clinical care settings in a multi-center trial.
**16**	Ensure representation of low-, middle-, and high-income countries in international multi-center trials on circadian-based interventions.	Ensure that international multi-center trials on circadian interventions represent a spectrum of healthcare models and economic settings, from low-income and high-income regions.

Table E3. Responses to question "Does this list of propositions and recommendations reflect the key points and scope of the discussions during the Lorentz workshop?"

Answer option	***n (%)***
Yes, completely	14 (61%)
Yes, but with some minor omissions	9 (39%)
No, it misses some important points	0 (0%)
No, it does not reflect the discussions at all	0 (0%)
